# Osteoking Decelerates Cartilage Degeneration in DMM-Induced Osteoarthritic Mice Model Through TGF-β/smad-dependent Manner

**DOI:** 10.3389/fphar.2021.678810

**Published:** 2021-06-15

**Authors:** Houfu Ling, Qinghe Zeng, Qinwen Ge, Jiali Chen, Wenhua Yuan, Rui Xu, Zhenyu Shi, Hanting Xia, Songfeng Hu, Hongting Jin, Pinger Wang, Peijian Tong

**Affiliations:** ^1^ The First College of Clinical Medicine, Zhejiang Chinese Medical University, Hangzhou, China; ^2^ Institute of Orthopaedics and Traumatology, the First Affiliated Hospital of Zhejiang Chinese Medical University, Hangzhou, China; ^3^ Department of Orthopaedics and Traumatology, Shaoxing Hospital of Traditional Chinese Medicine, Shaoxing, China; ^4^ College of Pharmaceutical Science, Zhejiang Chinese Medical University, Hangzhou, China; ^5^ Department of Orthopaedic Surgery, the First Affiliated Hospital of Zhejiang Chinese Medical University, Hangzhou, China

**Keywords:** osteoking, osteoarthritis, cartilage protection, conditonal knockout, TGF-β/Smad pathway

## Abstract

Osteoarthritis (OA) is a common disease characterized by cartilage degeneration. In recent years much attention has been paid to Traditional Chinese Medicine (TCM) since its treatments have shown efficacy for ameliorating cartilage degradation with mild side effects. Osteoking is a TCM prescription that has long been used in OA treatment. However, the exact mechanism of Osteoking are not fully elucidated. In the current study, destabilization of the medial meniscus (DMM)-induced OA mice was introduced as a wild type animal model. After 8 weeks of administration of Osteoking, histomorphometry, OARSI scoring, gait analysis, micro-CT, and immunohistochemical staining for Col2, MMP-13, TGFβRII and pSmad-2 were conducted to evaluate the chondroprotective effects of Osteoking *in vivo*. Further *in vitro* experiments were then performed to detect the effect of Osteoking on chondrocytes. TGFβRII^Col2ER^ transgenic mice were constructed and introduced in the current study to validate whether Osteoking exerts its anti-OA effects *via* the TGF-β signaling pathway. Results demonstrated that in wild type DMM mice, Osteoking ameliorated OA-phenotype including cartilage degradation, subchondral bone sclerosis, and gait abnormality. Col2, TGFβRII, and pSmad-2 expressions were also found to be up-regulated after Osteoking treatment, while MMP-13 was down-regulated. *In vitro*, the mRNA expression of MMP-13 and ADAMTS5 decreased and the mRNA expression of Aggrecan, COL2, and TGFβRII were up-regulated after the treatment of Osteoking in IL-1β treated chondrocytes. The additional treatment of SB505124 counteracted the positive impact of Osteoking on primary chondrocytes. In TGFβRII^Col2ER^ mice, spontaneous OA-liked phenotype was observed and treatment of Osteoking failed to reverse the OA spontaneous progression. In conclusion, Osteoking ameliorates OA progression by decelerating cartilage degradation and alleviating subchondral bone sclerosis partly *via* the TGF-β signaling pathway.

## Introduction

Osteoarthritis (OA) was a common disease of the cartilage that is characterized by cartilage degradation, synovial tissue inflammation, subchondral bone sclerosis, and osteophyte formation. (Wang et al., 2011; Bomer et al., 2015). The main risk factors of OA are aging, environmental factors, joint dysplasia and injury, and inherent genetic alternations (Felson, 2006; Martel-Pelletier et al., 2016; Roos and Arden, 2016; O'Brien and McDougall, 2019). Until recently, treatments for OA have mainly focused on symptom relief because no disease-modified osteoarthritis drugs are widely accepted to be effective in protecting cartilage from degradation (Martel-Pelletier et al., 2016).

TGF-β is a large family of growth factors that plays a crucial role in early embryonic development and postnatal growth and regulation of cell differentiation, apoptosis, and migration in different tissues (Gordon and Blobe, 2008). The chondrocytes played an important role in maintaining the homeostasis of cartilage (Buckwalter et al., 2005), the dysregulation of which was directly linked to the pathological development of OA (Buckwalter and Brown, 2004; Brandt et al., 2009). TGF-β signaling also played an important role in the regulation of cartilage, which was responsible for regulating the synthesis and degradation of extracellular matrix proteins, controlling the proliferation and differentiation of chondrocytes, and inhibiting the hypertrophy and maturation of chondrocytes (Dünker and Krieglstein, 2000; Blaney Davidson et al., 2007; Jin et al., 2011; Zhen et al., 2013; Xie et al., 2016). The mice with global knockout of Smad3 gene exhibited spontaneous OA-liked phenotype characterized by chondrocytes hypertrophy with the upregulated expression of COL10, progressive loss of articular cartilage, and osteophytes formation (Yang et al., 2001). Moreover, deletion of the TGF-β receptor type II gene in adult mice chondrocytes has shown OA-liked phenotype with the upregulation of Runx2, MMP13, ADAMTS5, and Col10 (Shen et al., 2013, 2014). In addition, OA-liked pathological changes were significantly alleviated in TGFβRII and MMP-13 double-knockout mice. Treatment of TGFβRII^Col2ER^ mice with an MMP-13 inhibitor also slowed OA progression. Collectively, these studies demonstrate that MMP-13 is a critical downstream target gene involved in the TGF-β signaling pathway during the development of OA (Shen et al., 2013).

Much attention had been attracted to Traditional Chinese Medicine (TCM) in recent years (Li H. et al., 2017). Several classical TCM formulas such as the Bu-Shen-Huo-Xue formula (Wang et al., 2018) and Du-Huo-Ji-sheng decoction (Wu et al., 2013; Liu et al., 2014) have been proven to ameliorate OA progression by protecting cartilage from degradation. Osteoking, a classical TCM formula, also known as the Heng-gu-gu-Shang-Yu-He-Ji, originated from the ethnic Yi in the Yunnan province of China and was approved by the Chinese State Food and Drug Administration in 2002. Osteoking is composed of several Chinese herbs, including Pericarpium Citri Reticulatae, Flos Carthami, Radix Notoginseng, Cortex Eucommiae, Radix Ginseng, Radix Astragali, Flos Daturae, Schizophragma integrifolium, and trionyx sinensis carapace. It has long been used in treating OA; however, the precise mechanisms were still not well understood.

This study hypothesized that Osteoking could attenuate cartilage degeneration and ameliorate subchondral bone sclerosis. Firstly, the effect of Osteoking on cartilage and subchondral bone was evaluated in DMM-induced OA mice. Then, the impact of Osteoking on chondrocytes was detected. To further determine the possible mechanism, TGFβRII conditional knockout (cKO) in cartilage mice (TGFβRII^Col2ER^ mice) was then generated to validate whether Osteoking exerts therapeutic effects *via* the TGF-β signaling pathway.

## Materials and Methods

### Preparation and UPLC Analysis of Osteoking

**FIGURE 1:**
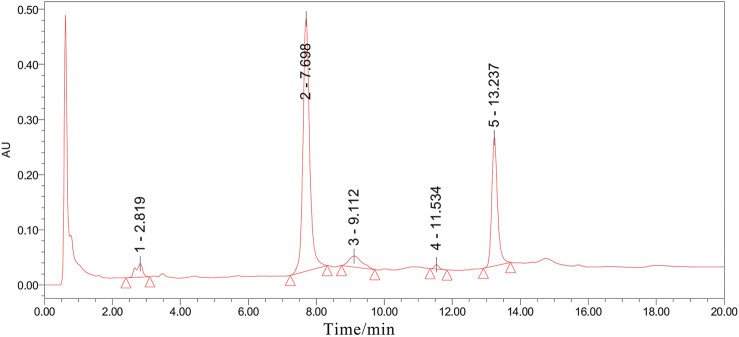
The UPLC analysis result of Osteoking solution. All chromatographic peak signaling was recorded at 260 nm and the peak area was integrated under the instrument’s protocol. Peak 1–5 respectively indicated Astragaloside, Aucubin, Ginsenoside, Notoginsenoside, and Hesperidin.

All Osteoking used in this study were purchased from the Yunnan Crystal Natural Pharmaceutical Co., Ltd. (Kunming, China) (lot. No. 20190330). The relative proportions are shown in Table 1. The identification of all the plant materials used in this study was undertaken by Yunnan Crystal Natural Pharmaceutical Co., Ltd. according to the Chinese Pharmacopeia (2015, Edition). Ultra-Performance Liquid Chromatograph (UPLC) was utilized to control the quality of Osteoking and identify the accurate chemical component. Five chromatogram peaks that represent five drug monomers respectively were identified in UPLC and showed in Figure 1.

**TABLE 1:** The composition of Osteoking.

Chinese herb	Full taxonomy name	Latin name	Wight(g)	Parts used
Chen pi	Citrus reticulata blanco	Pericarpium citri reticulatae	30	Peel
Hong hua	Carthamus tinctorius L	Flos carthami	30	Flower
San qi	Panax notoginseng (burkill) F.H.Chen	Radix notoginseng	30	Root
Du zhong	Eucommia ulmoides oliv	Cortex eucommiae	30	Bark
Ren shen	Panax ginseng C.A.Mey	Radix ginseng	20	Root
Huang qi	*Astragalus* mongholicus bunge	Radix astragali	40	Root
Yang jin hua	Datura metel L	Flos daturae	15	Flower
Zuan di feng	Schizophragma integrifolium (Franch.)Oliv	Schizophragma integrifolium	25	Root; Stem
Bie jia	Carapax trionycis	Trionyx sinensis carapace	10	Carapace

### Mice

In this study, 10-week-old C57BL/6J male mice for the osteoarthritic model were purchased from the Experimental Animal Center of Zhejiang Chinese Medical University (Hangzhou, China). The Col2-CreER mice and TGFβRII^flox/flox^ mice were donated from Rush University Medical Center (Chicago, United States). TGFβRII^flox/flox^ mice were bred with Col2-CreER mice to generate cartilage TGFβRII conditional knockout mice (TGFβRII^Col2ER^ mice). Transgenic mice genotyping was identified by PCR with a DNA extraction kit (Sigma, St. Louis, MO, United States) from tail biopsy (Shen et al., 2013; Wang et al., 2018). TGFβRII^flox/flox^ mice were used as Cre-negative controls. Both Cre-negative mice and TGFβRII^Col2ER^ mice were induced for five consecutive days at 2-week-old with Tamoxifen (100 mg/kg) by intraperitoneal injection. Only male transgenic mice were used for further experiments to avoid gender-dependent differences.

### Osteoarthritic Model

Destabilization of the medial meniscus (DMM) was undertaken on 10-week-old male C57BL/6J mice to construct the knee osteoarthritic model, as outlined in a previous report (Culley et al., 2015). Briefly, DMM surgery was performed on the right hind limb of WT mice. First, a 3 mm longitudinal incision was made on the medial part of the knee under anesthesia, blunt dissection of the knee extensor muscles and patellar ligament were performed to expose the medial meniscotibial ligament (MMTL); Then, the MMTL was transected to make destabilization of the medial meniscus (DMM); At last, the medial joint capsule was sutured, and the skin was closed. The sham surgery was also performed by a similar surgical approach without manipulating the joint tissue in WT mice.

All C57BL/6J mice underwent DMM surgery or sham surgery. They were then randomly divided into three groups: 1) sham group; 2) DMM group; 3) Osteoking group. In transgenic mice, the TGFβRII^flox/flox^ mice were used as Cre-negative controls. All transgenic mice were divided into three groups: 1) Cre-negative group; 2) TGFβRII^Col2ER^ group; 3) TGFβRII^Col2ER^ + Osteoking group. 10 mice were in each group. Osteoking was orally administered to both the Osteoking group and TGFβRII^Col2ER^ + Osteoking group once a day with a dose of 3.79 ml/kg body weight for eight consecutive weeks. Dosages administered were determined according to human-mouse equivalent dosage conversion. The other groups were treated with the same doses of normal saline by oral gavage. All mice were free for food and water in this study and all studies were approved by the Committee on the Ethics of Animal Experiments of Zhejiang Chinese Medical University (LZ12H27001).

### Gait Analysis

The gait of WT mice was recorded and analyzed using a DigiGait imaging system (Mouse Specifics) at the eighth week after treatment. Briefly, mice ran on a transparent flat treadmill at a specific speed (17 cm/s), while a video camera captured ventral images. Animals ran for a maximum of 30 s for each measurement, with segments of 5 s (which more than 10 consecutive strides) used for analysis. The following indexes of the right hind limb (surgical one) were observed: paw area, stride length, swing, and stance.

### Micro-CT Analysis

All mice were sacrificed and the right knee joints were harvested at week eight post-operation. Then Micro-computed tomography (Micro-CT) (Skyscan 1176, Bruker μCT, Kontich, Gelgium) was used to analyze the knee joints. The area between the proximal tibia growth plate and the tibial plateau was chosen as the region of interest and the parameters collected by Micro-CT were Percent bone volume (BV/TV, %), Trabecular thickness (Tb. Th, mm), and Bone mineral density (BMD, g/cm^3^).

### Histological Analysis

After micro-CT analysis, the samples were successively fixed in 4% paraformaldehyde for 3 days, decalcified with 14% EDTA solution for 14 days, and subsequently embedded in paraffin. Then 3-μm thick sections at the medial compartment of the joints were cut for Alcian Blue Hematoxylin/Orange G staining and Toluidine Blue staining to analyze the gross cartilage structural changes. Histomorphometric analysis was performed through OsteoMeasure software (Decatur, GA). Cartilage structure degeneration was scored by three blinded observers according to the recommendations of the Osteoarthritis Research Society International (OARSI).

### Immunohistochemistry

The immunohistochemistry was examined to observe the expressions of type II collagen (Col2), matrix metalloproteinase 13 (MMP-13), TGF-β receptor type II (TGFβRII) and phosphorylated protein mothers against decapentaplegic homolog 2 (pSmad2) in cartilage. Briefly, the deparaffinized sections were soaked in 0.3% hydrogen peroxide to block the activity of endogenous peroxidase, then blocked with normal goat serum (diluted 1:20) for 20 min at room temperature. Subsequently, the primary antibodies were added and incubated overnight at 4°C. The next day, the sections were treated with secondary antibodies for 30 min and positive staining was visible by using diaminobenzidine solution (Invitrogen, MD, United States). Then counterstaining was performed with hematoxylin for 5 s. Anti-Col2 (Abcam, ab34712, 1:200), anti-TGFβRII (Santa, sc400, 1:200), anti-pSmad2 (Abcam, ab188334, 1:100), anti-MMP-13 (Abcam, ab39012, 1:100) were used in this study.

### Preparation of Drug Serum

Fifty 12-week-old male rats (200 ± 30 g) were purchased from the Experimental Animal Center of Zhejiang Chinese Medical University and randomly divided into two groups. Rats in the Osteoking group were treated with Osteoking (2.625 ml/1 kg body weight) for seven consecutive days while the rats in the control group were administrated with normal saline. All rats were euthanatized for blood sampling and the serum was collected by centrifugation at 3,000 rpm/min. The serum was then filtered and inactivated at 56°C for 30 min, and stored at −80°C.

### Cell Isolation and Culture

Primary rat chondrocytes were obtained from the femoral head of 2-week-old rats purchased from the Experimental Animal Center of Zhejiang Chinese Medical University. Rats were sacrificed and disinfected with 75% ethyl alcohol. Specimens were isolated and rinsed by Phosphate Buffer Saline (PBS) 3 times. Then the cartilage tissues were digested with a culture medium, consisted of collagenase *p*, DMEM/F-12, and 10% fetal bovine serum (FBS) at 37°C for 4 h. Chondrocytes were cultured in DMEM/F-12 medium containing 10% FBS and 1% streptomycin/penicillin in 5% CO_2_ at 37°C for further experiment.

### Cell Viability Assay

Cell counting kit-8 (CCK-8) assay was executed according to the manufacturer instructions to detect the cytotoxic effect of Osteoking on primary rat chondrocytes. Chondrocytes were seeded in 96-well plates with a density of 10^4^ cells per well. Then, chondrocytes were treated with drug serum of Osteoking at different dosages for 24 and 48, using the (%) mean percentage of Osteoking contained in culture medium. The CCk-8 reagents were then added to each well and chondrocytes were incubated for another 2 h. The absorption of each well was detected by the microplate reader at 450 nm finally.

### Quantitative RT-PCR

The total RNA in all primary rat chondrocytes was extracted by TRlzol reagent. Reverse transcription was then carried out with a cDNA Synthese Kit (Bmake, B24408, Beijing). The quantitative real-time-polymerase chain reaction (qRT-PCR) was conducted with SYBR Premix Ex Taq™ II (Takara, Dalian, China). Quantitative analysis was performed using QuantStudio™ Real-Time PCR Software. β-actin was regarded as the reference gene for quantitative analysis. The primer sequences of the target gene used in the current study are shown in Table 2.

**TABLE 2:** Primer sequences for quantitative RT-PCR.

Gens	Primer sequences	
β-actin	Forward	CAC​TAT​CGG​CAA​TGC​GGT​TCC
	Reverse	CAG​CAC​TGT​GTT​GGC​ATA​GAG​GTC
TGFβRII	Forward	CAC​CTC​CAT​CTG​TGA​GAA​GCC
	Reverse	GGC​AAA​CGG​TCT​CCA​GAG​TAA
MMP-13	Forward	AAC​CAA​GAT​GTG​GAG​TGC​CTG​ATG
	Reverse	CAC​ATC​AGA​CCA​GAC​CTT​GAA​GGC
ADAMTS5	Forward	TCC​TCT​TGG​TGG​CTG​ACT​CTT​CC
	Reverse	TGG​TTC​TCG​ATG​CTT​GCA​TGA​CTG
Aggrecan	Forward	GAT​CTC​AGT​GGG​CAA​CCT​TC
	Reverse	TCC​ACA​AAC​GTA​ATG​CCA​GA
COL2	Forward	CTC​AAG​TCG​CTG​AAC​AAC​CA
	Reverse	GTC​TCC​GCT​CTT​CCA​CTC​TG

### Statistical Analysis

All data were presented as mean ± standard deviation. Comparing the mean among groups, we used one-way analysis of variance (ANOVA) test by Tukey’s test. A *p* value of <0.05 was considered statistically significant. The statistical analysis was performed using SPSS 23.0 software.

## Results

### Osteoking Decelerated the OA Progression in DMM-Induced Osteoarthritic Mice

**FIGURE 2:**
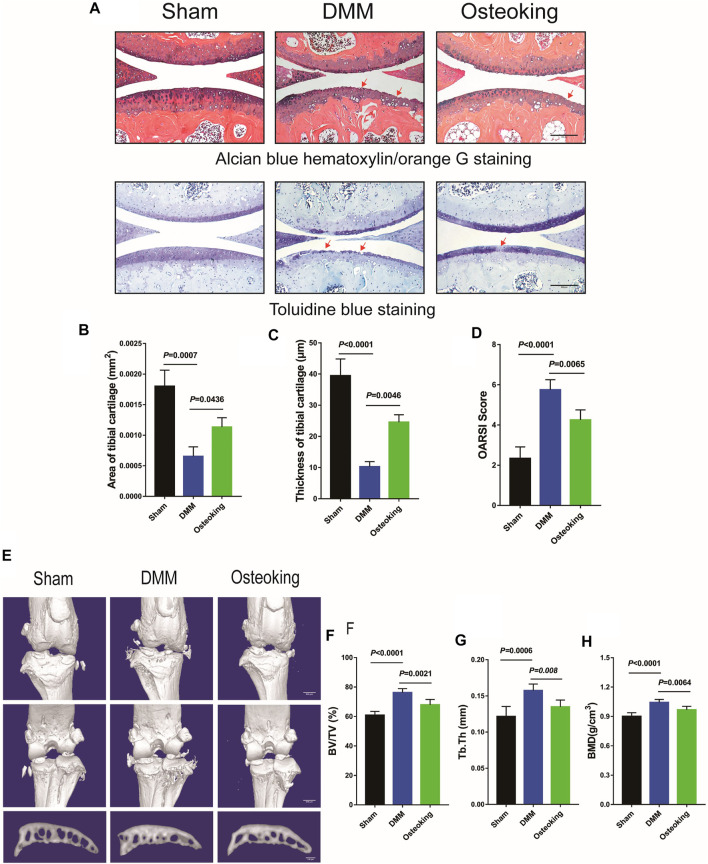
Osteoking decelerated OA progression in a DMM-induced osteoarthritic model. **(A)** Alcian blue hematoxylin/orange G staining and Toluidine blue staining of the right knee joint in WT mice (surgical one). Red arrows indicated the area of cartilage erosion. Scale bar = 100 μm. Morphological quantitative analysis of **(B)** area of tibial cartilage (mm^2^) and **(C)** thickness of tibial cartilage (μm). **(D)** OARSI scoring of the sections analyzed by histomorphometry. Micro-CT was performed to analyze the changes in bone structure. **(E)** Representative 3D reconstruction of the right knee joint and subchondral bone. Quantitative analysis of the subchondral bone **(F)** BV/TV (%), **(G)** Tb.Th (mm) and **(H)** BMD (g/cm^3^). Data were presented as means ± SD (n = 10). The exact *p* value is presented in the corresponding graph.

In order to determine whether Osteoking had a protective effect on the cartilage in OA, Alcian blue hematoxylin/orange G staining and Toluidine blue staining were performed to observe the cartilage changes in DMM-induced mice. As shown in Figure 2A, prominent focal cartilage defects were observed in the DMM-induced model mice and the Osteoking treated mice showed more intact cartilage tissue, which demonstrated that Osteoking could dramatically protect the cartilage from degeneration in OA progression. Quantitative analysis was performed by calculating the proportion and thickness of cartilage and results showed that both the area and thickness of cartilage were found to be significantly reduced in DMM-induced OA mice, and Osteoking treatment significantly reversed the trend (Figures 2B,C). Subsequently, significantly higher scores were found in the DMM group compared to the sham group through the analyses of the histological scoring system recommended by OARSI. Accordingly, the Osteoking group showed significantly lower scores (Figure 2D).

For further analysis of micro-construction changes of subchondral bone, interior subchondral bone was scanned through micro-CT and subsequently quantitatively analyzed by CTan software. The Percent bone volume (BV/TV), Trabecular thickness (Tb. Th), and Bone mineral density (BMD) of subchondral trabecular bone were analyzed respectively. As shown in Figure 2E, a small sized osteophyte formation was observed at the margin of joint in DMM mice, meanwhile, there was no distinctive osteophyte observed in the sham and Osteoking group. Significant increase of BV/TV, Tb.Th and BMD revealed obvious subchondral bone sclerosis in DMM-induced OA mice. Interestingly, Osteoking treatment significantly reversed the trend in BV/TV, Tb.Th and BMD (Figures 2F–H), suggesting that Osteoking treatment alleviated the subchondral bone sclerosis compared to the DMM group. Taken together, Osteoking treatment possessed anti-OA effects by protecting cartilage from degradation and subchondral bone from sclerosis in wild type DMM-induced OA mice.

### Osteoking Upregulated the Expression Level of TGFβRII and pSmad2 in the Cartilage of DMM-Induced Osteoarthritic Mice

**FIGURE 3:**
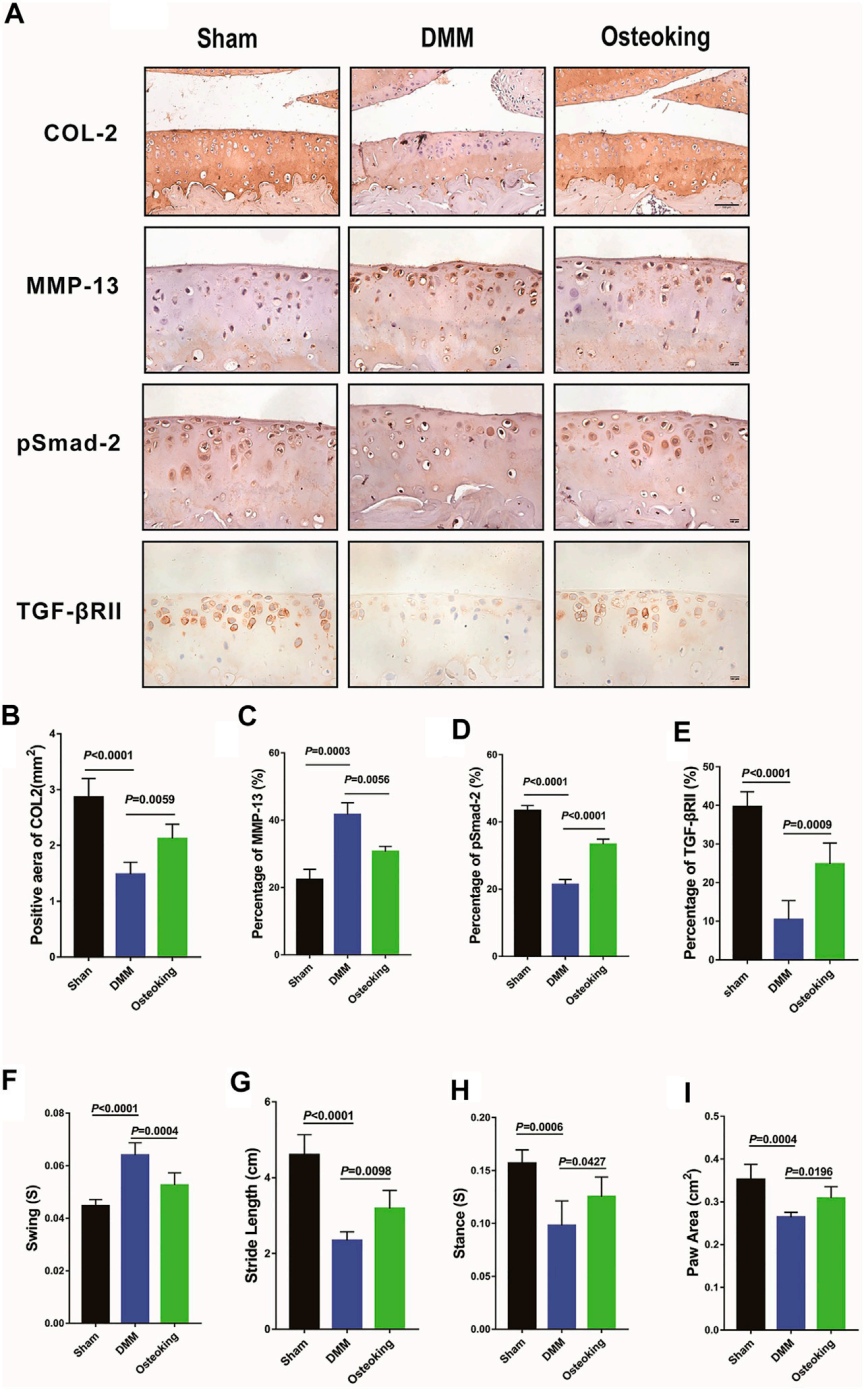
Osteoking up-regulated the expression of TGFβRII and pSmad2 in DMM-induced WT osteoarthritic mice. **(A)** immunohistochemical staining of Col2, MMP-13, and pSmad2 and TGFβRII in cartilage. Scale bar = 100 μm. **(B–E)** Quantification of the positive repression area of Col2 (mm^2^), Percentage of positive expression of pSmad2, TGFβRII, and MMP-13 (%). Gait analysis was performed by DigiGait imaging system. **(F)** Swing (s), **(G)** Stride length (cm), **(H)** Stance (s), **(I)** Paw area (cm^2^) of the right hind limb were chosen as the observation index. The Data are presented as means ± SD (n = 10). The exact *p* value is presented in the corresponding graph.

An imbalance between anabolism and catabolism leads to cartilage degradation in OA. Catabolic genes such as MMP-13 are up-regulated in OA progression. A key molecule in TGF-β signaling, pSmad-2, which plays a critical role in the regulation of cartilage, was down-regulated in OA progression. Meanwhile, Col2, a representative marker of cartilage metabolic activity, was down-regulated in OA progression. In the current study, IHC staining was conducted to determine whether Osteoking restore the balance between anabolism and catabolism in cartilage. Results demonstrated that Col2, TGFβRII, and pSmad2 were significantly down-regulated while MMP-13 was up-regulated in DMM-induced OA mice. Treatment of Osteoking significantly reversed the trend by improving the expression level of Col2, TGFβRII, and pSmad2 in cartilage and reducing the expression of MMP-13 in chondrocytes (Figure 3A). Quantitative analysis of IHC staining was done and results agreed with the alterations showed in Figures 3B–E. The results indicated that Osteoking protects cartilage by maintaining the metabolism balance partly *via* the TGF-β/smad2 signaling pathway.

### Osteoking Ameliorated the Gait Abnormality of the DMM-Induced Osteoarthritic Mice

Gait disturbance was reported in DMM-induced OA mice since the pain and dysfunction of the knee joint. In the current study, gait analysis was conducted to determine whether Osteoking alleviates the symptoms in DMM-induced OA mice. As shown in Figures 3F–I, compared with the sham mice, decreases of paw area, stride length, and stance and increases in swing were observed in DMM mice, and the treatment of Osteoking increased the paw area, stride length, stance and reduced the swing. Results revealed Osteoking improved the function of the knee joint and alleviated the DMM-induced gait disturbance.

### Osteoking Inhibited the Catabolism and Promoted Anabolism of Primary Rat Chondrocytes Treated With IL-1β *in vitro*

**FIGURE 4:**
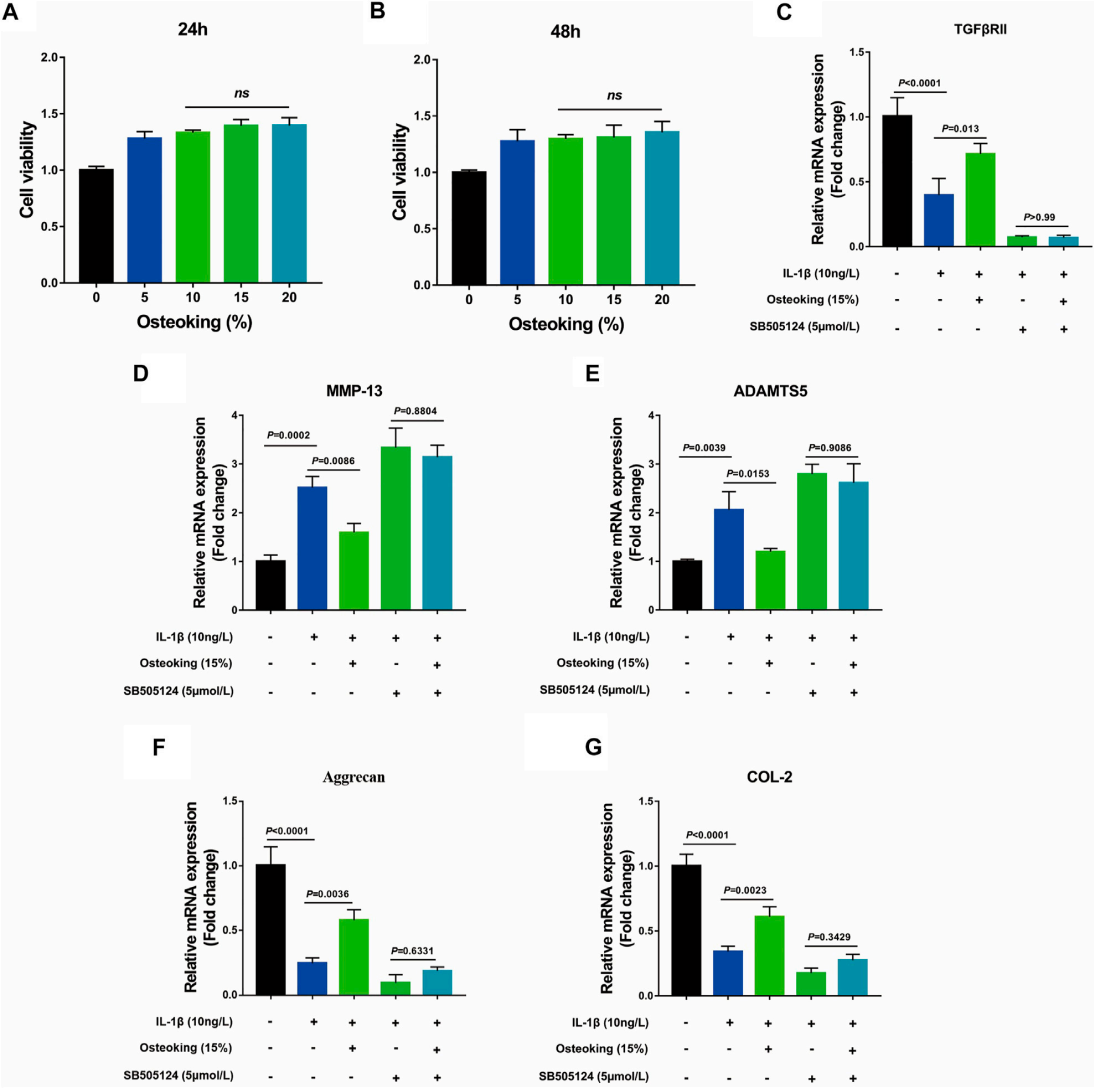
The effect of Osteoking on IL-1β-induced primary rat chondrocytes *in vitro*. **(A–B)** Cell viability of primary rat chondrocytes cultured with various concentrations of Osteoking for 24 and 48 h **(C–G)** The gene expression of IL-1β-induced primary rat chondrocytes was treated with 15% Osteoking and TGF-β inhibitor (SB505124) for 24 h. The Data are presented as means ± SD (n = 3 in each group). The exact *p* value is presented in the corresponding graph.

Based on prior findings that Osteoking could protect cartilage from degradation *in vivo*, primary rat chondrocytes were utilized to explore the protective effect of Osteoking on chondrocytes. The results of CCK-8 showed that Osteoking had no impact on the viability of primary chondrocytes (Figures 4A,B). Furthermore, no significant difference was observed at the Osteoking content of 10–20% (Figures 4A,B), thus 15% Osteoking was used for further research. In addition, SB505124, an inhibitor of TGF-β, was used to validate the role of the TGF-β signaling pathway in the protective effect of Osteoking. Our data showed that Osteoking could significantly decrease the mRNA expression of MMP-13 and ADAMTS5 (Figures 4D,E), increased the mRNA expression of Aggrecan and COL2 (Figures 4F,G), indicated that Osteoking could inhibit the catabolism and promote the anabolism activities of chondrocytes, which were consistent with the IHC staining. In addition, Osteoking treatment could up-regulate the mRNA expression of TGFβRII reduced by IL-1β treatment (Figure 4C). However, Osteoking seems to cause demise in the chondroprotective function in IL-1β and SB505124 co-treated primary chondrocytes (Figures 4D–G). These results demonstrated that Osteoking could promote anabolism and protect chondrocytes from catabolism partly *via* the TGF-β signaling pathway.

### Osteoking Could Not Decelerate OA progression in TGFβRII^Col2ER^ Mice

**FIGURE 5:**
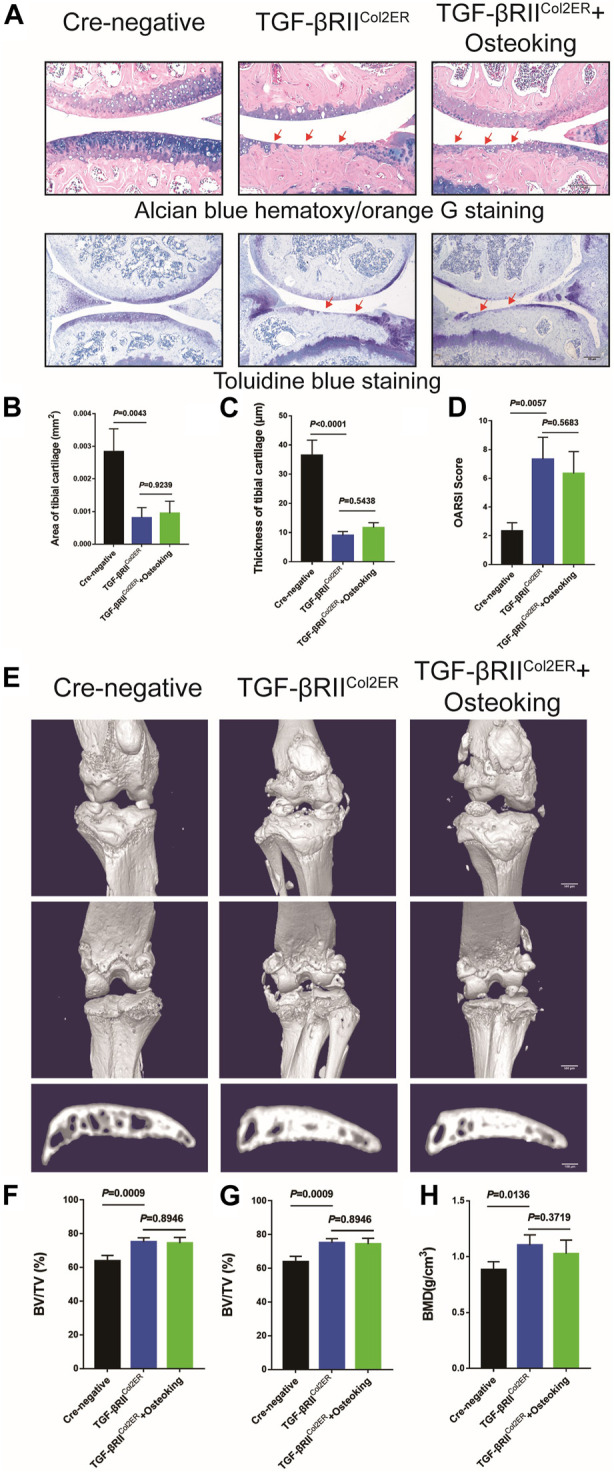
The cartilage protection effect of Osteoking was lost in TGFβRII^Col2ER^ mice. **(A)** Alcian blue hematoxylin/orange G staining and Toluidine blue staining of the tight knee joint in TGFβRII^Col2ER^ mice. Red arrows indicate the area of cartilage erosion. Scale bar = 100 μm. Morphological quantitative analysis of **(B)** area of tibial cartilage (mm^2^) and **(C)** thickness of tibial cartilage (μm). **(D)** OARSI scoring of the sections analyzed by histomorphometry. **(E)** Representative 3D reconstruction of the right knee joint and subchondral bone. Quantitative analysis of the subchondral bone of right knee joints **(F)** BV/TV (%), **(G)** Tb.Th (mm) and **(H)** BMD (g/cm^3^) were chosen as the observation indexes. Data are presented as means ± SD (n = 10). The exact *p* value is presented in the corresponding graph.

For further validation of the mechanism of Osteoking in treating OA, TGFβRII^Col2ER^ mice were generated as previously described. TGFβRII^Col2ER^ mice exhibited severe spontaneous OA-liked phenotype, including cartilage degradation, subchondral bone sclerosis. ABH staining and Toliudine Blue staining were conducted to assess cartilage destruction. As shown in Figure 5A, overt cartilage erosion and subchondral bone sclerosis were observed in TGFβRII^Col2ER^ compared with the Cre-negative mice. furthermore, no obvious alleviation of the TGFβRII^Col2ER^ spontaneous OA-liked phenotype was found after the treatment of Osteoking. Cartilage proportion and thickness were also calculated and no significant differences were found between TGFβRII^Col2ER^ mice and Osteoking treated TGFβRII^Col2ER^ mice (Figures 5B,C). OARSI score was consistent with the above-mentioned results (Figures 5D).

In addition, micro-CT was conducted to evaluate the osteophyte formation and subchondral bone sclerosis. As shown in Figure 5E, significant osteophyte formation and subchondral bone sclerosis were found in TGFβRII^Col2ER^ mice compared with the Cre-negative group. Reflecting the assessment results relating to cartilage in TGFβRII^Col2ER^ mice, no significant chanes were found after treatment with Osteoking (Figures 5F–H). Taken together, conditional deletion of TGF-β receptor II in chondrocytes counteracted the anti-OA effects of Osteoking in DMM mice, suggesting that Osteoking exerts its cartilage protective effect partly *via* the TGF-β signaling pathway.

### Osteoking Could Not Ameliorate the Upregulation of MMP-13 in TGFβRII^Col2ER^ Mice

**FIGURE 6:**
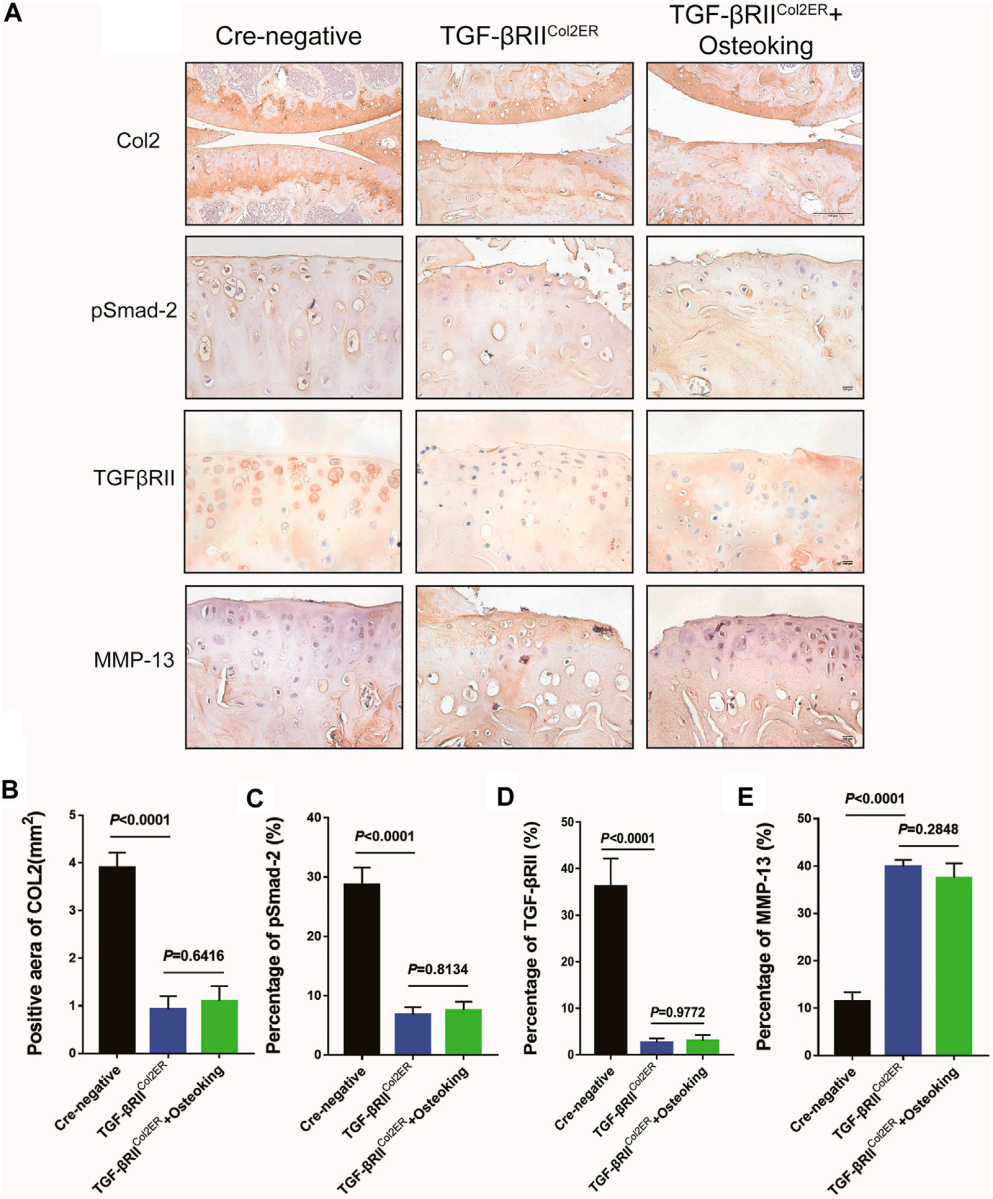
The expression of MMP-13 was not down-regulated after the treatment of Osteoking in TGFβRII^Col2ER^ mice. **(A)** Immunohistochemistry staining for Col2, pSmad2, TGFβRII, and MMP-13 in cartilage. Scale bar = 100 μm. **(B–E)** Quantification of the positive repression of these markers. Data are presented as means ± SD (n = 10). The exact *p* value is presented in the corresponding graph.

The expressions of MMP-13, TGFβRII, pSmad2, and Col2 were also detected in TGFβRII^Col2ER^ mice. As shown in Figures 6A,C,D, the expression of TGFβRII and pSmad2 in TGFβRII^Col2ER^ mice had reduced more than 85% compared with Cre-negative mice, indicating that the TGF-β pathway was successfully blocked in this study. As expected, the treatment of Osteoking did not contribute to protecting Col2 from degradation in TGFβRII^Col2ER^ mice (Figures 6A,B). The down-regulated expression of the MMP-13 was not observed in TGFβRII^Col2ER^ mice treated with Osteoking (Figures 6A,E).

## Discussion

Recent conventional treatments of OA have focused on expectant treatment and artificial joint replacement. Joint pain is one of the most prominent symptoms of OA, thus painkiller drugs, such as non-steroidal anti-inflammatory drugs (NSAIDs), non-specific cyclooxygenase inhibitors, selective cyclooxygenase-2 (COX-II) inhibitors, opiates, and non-opioid oral analgesics are widely applied in clinical settings (Vaishya et al., 2016). However, the long-term use of these drugs might induce side effects, for example, an adverse reaction in the gastrointestinal tract. Furthermore, therapies for these symptoms just relieved OA and had no effective intervention on OA progression.

Traditional Chinese Medicine (TCM), as the accumulation of thousands of Chinese civilizations, had attracted much attention on its prominent treatment of OA. Besides its therapeutic effectiveness, it was also characterized by fewer adverse effects. Some studies have demonstrated that parts of TCM (Liu et al., 2014; Wang et al., 2018; Xia et al., 2020), such as Duo Huo JI Sheng Decoction (DHJSD), Bushen Huoxue Decoction (BSHXD), and Jiawei Yanghe Decoction (JWYHD), could ameliorate OA progression and protect articular chondrocytes. Furthermore, the possible mechanisms of these TCM were analyzed. Osteoking, also known as Heng-Gu-Gu-Shang-Yu-He-Ji, originated from the Yi people in Yunnan Province of China. It possesses the function of promoting blood circulation, nourishing the liver and kidney according to Chinese Traditional Medicine. Based on the UPLC analysis, five drug monomers were identified, including Astragaloside, Aucubin, Ginsenoside, Notoginsenoside, and Hesperidin. Previous reports had reported that Hesperidin (Lin et al., 2020), extracted from Pericarpium Citri Reticulatae, attenuated cartilage destruction and reduced IL-1β and TNF-α levels in a surgically-induced OA model. Aucubin (Wang et al., 2015, 2019) a natural compound isolated from Cortex Eucommiae, had a protective effect on cartilage, slowing OA progression in the OA mice model. These reporters might account for the therapeutic function of Osteoking in OA. Accordingly, the positive effect of Osteoking on cartilage protection was observed in DMM mice in the current study. Firstly, the DMM model, as a gold standard in OA models (Glasson et al., 2007; Culley et al., 2015), was performed in 10-week-old male WT mice to duplicate OA progression. Then, remarkable cartilage degeneration and subchondral bone sclerosis were observed through histological and micro-CT analysis, indicating the successful establishment of the OA model. In addition, better cartilage and subchondral bone construction were exhibited in Osteoking treated DMM mice, suggesting the protective function of Osteoking during the development of OA.

In healthy cartilage, the ECM was kept in a slow, continuous state of turnover, often described as homeostasis. When the ECM was in homeostasis, overall anabolic and catabolic activities of matrix synthesis and degradation were in balance (Rahmati et al., 2017; Guilak et al., 2018; Krishnan and Grodzinsky, 2018; Peng et al., 2020). However, the homeostasis of ECM was disrupted during the development of OA, characterized by the increase of catabolic activities, caused the gradual degeneration of cartilage (Guilak et al., 2018). The articular chondrocytes played an important role in maintaining the homeostasis of cartilage and dysregulation of chondrocytes was directly linked to the pathological development of OA (Buckwalter and Brown, 2004; Buckwalter et al., 2005; Brandt et al., 2009). The disordered chondrocytes would cause the degradation of ECM and down-regulated protein level of Col2, proteoglycan, etc. (Aigner et al., 1997; Shlopov et al., 1997; Shiomi et al., 2010). Accordingly, the expression of MMP-13, a major enzyme that degrades Col2, proteoglycan in cartilage, was up-regulated significantly (Wang et al., 2013; Li L. et al., 2017). *In vivo*, the immunohistochemical staining was utilized to analyze the protein expression of Col2 and MMP-13 in cartilage, data showed the significant down-regulation of Col2 and up-regulation of MMP-13 in DMM mice, which was in line with previous reports. Furthermore, we found that Osteoking treatment significantly reversed the Col2 and MMP-13 expression tendency in DMM mice, indicated that Osteoking could maintain the ECM homeostasis to protect cartilage partially through inhibiting the MMP-13. And interestingly, the expression of TGFβRII and pSmad2 were up-regulated in Osteoking treated DMM mice. *In vitro*, primary mice chondrocytes were gained to explore the effect of Osteoking on chondrocytes. Osteoking showed a significant protective effect on chondrocytes, which was reflected in decreasing the mRNA expression of MMP-13 and ADAMTS5 and up-regulating the mRNA expression of Aggrecan and COL2. And interestingly, the mRNA expression of TGFβRII was significantly up-regulated in Osteoking treated primary chondrocytes compared with IL-1β treated only. What’s more, additional treatment of SB505124, a chemical inhibitor of TGF-β, counteracted the chondroprotective effect of Osteoking in primary rat chondrocytes. Collectively, the protective effect of Osteoking on cartilage and chondrocytes was validated and suggested that TGF-β played an important role during the working process.

In order to gain better insights into the effects of Osteoking in OA progression, we analyzed the gait difference of DMM-induced model WT mice with or without the treatment of Osteoking. Active and passive gait analysis had been used in various laboratory models on pain analysis (Fuseya et al., 2016; Slotkin et al., 2016), such as peripheral inflammatory, neuropathic, and cancer pain. Some studies have demonstrated that pain and dysfunction would result in gait abnormality in the DMM-induced model. Analysis of Col10-deficient mice that develop OA spontaneously revealed reduced stride length and increased swing time compared to wild-type mice (Costello et al., 1985). The induction of OA in TGF-β1-injected mice by treadmill running also led to increased swing time and decreased stance time (Plaas et al., 2011). Paw area was decreased in rodents with carrageenan-induced rheumatoid arthritis (Culley et al., 2015). Our observations were consistent with previous research, which might be due to two reasons: one is that Osteoking could promote blood circulation, as discussed in TCM theory, and it might work as a painkiller drug; the other one is Osteoking could ameliorate the cartilage degeneration in DMM mice as we showed above, effectively ameliorated the dysfunction of OA. However, the exact effect and mechanism of Osteoking on gait improvement in DMM mice requires further exploration.

As prior research has demonstrated, Osteoking possesses an anti-osteoporosis effect by up-regulating the expression of osteogenesis-related genes and down-regulating the expression of TRAP (Qin et al., 2019). The study also demonstrated that Osteoking could promote bone healing in the necrotic femoral head of New Zealand white rabbits (Zhao et al., 2006). In this study, the subchondral bone sclerosis was significantly reduced in Osteoking treated DMM mice, which reflected the results of previous studies. Cartilage and subchondral bone are two important factors in OA progression, and much attention has been paid to the role of subchondral bone during OA progression, in recent years (Zhu et al., 2019). Previous reports demonstrate that microstructural alterations happen in the subchondral bone of the OA joint, including early-stage bone loss, late-stage bone sclerosis, and histopathological alterations (Li et al., 2013).

Subchondral bone plays an important role in load resorption and structural support and adapts to mechanical forces by bone remodeling. Noticeably, bone remodeling was altered during the development of OA because of the activation or inactivation of osteoclast-mediated resorption activity (Hu et al., 2020a). Furthermore, bone remodeling was involved in the coupling of osteoclastic bone resorption and osteoblastic bone formation to replace damaged with new bone (Hu et al., 2020b). In early-stage OA, the subchondral bone turnover was increased and characterized by activation of osteoclastic bone resorption (Maerz et al., 2016). As a result, the subchondral bone plate became thinner and more porous. Subchondral trabeculae were damaged and characterized by increased trabecular separation and decreased trabecular thickness. These changes cause the microdamage of subchondral bone in early-stage OA. Osteoking treatment started on the second day after DMM operation and indicated the intervention initiated in early-stage OA. According to its function promoting bone repair and inhibiting osteoclastic activation, Osteoking might inhibit the osteoclast-mediated subchondral bone resorption and promote the repair of subchondral bone microdamage. In addition, mechanical loads play an important role during the development of OA (Neogi et al., 2010). The impaired cartilage transfers excessive mechanical loads to the subchondral bone, which stimulates microstructural alterations in the subchondral bone (Hu et al., 2020a). However, Osteoking showed a significant protective effect on cartilage, which might partially guard the subchondral bone *via* reducing mechanical load transmission. The precise effect mechanism of Osteoking on subchondral bone needs to be further explored.

The chondroprotective effect of Osteoking was validated in DMM mice *in vitro* and *in vivo*. However, we still lack an understanding of the potential mechanism of Osteoking decelerating OA progression. Based on the up-regulated expression level of TGFβRII and pSmad2 in the cartilage of the Osteoking treated group, we preliminary predicted that the TGF-β pathway would join in the chondroprotective effect of Osteoking. To better understand the role of the TGF-β pathway in the functional process of Osteoking, mice with TGFβRII conditional knockout in cartilage (TGFβRII^Col2ER^ mice) were used to determine whether Osteoking still had a positive effect on cartilage with the blocking of TGF-β signaling. A previous report verified that special deletion of TGF-β receptor type II gene in adult mice chondrocytes showed OA-liked phenotype (Yang et al., 2001; Jin et al., 2011; Shen et al., 2014), with the up-regulation of Runx2, MMP13, ADAMTS5, and Col10. However, the OA-liked pathological changes were significantly alleviated in TGFβRII and MMP13 double-knockout mice. In addition, treatment of the MMP-13 inhibitor showed a similar result. This suggests that MMP-13 is a crucial downstream target gene involved in TGF-β signaling during the development of OA. In the current study, the expression level of pSamd2 and TGFβRII in the cartilage of TGFβRII^Col2ER^ mice was detected to confirm the efficiency of TGFβRII deletion. As shown in Figure 6A, more than 85% reduction was observed in TGFβRII^Col2ER^ mice compared with Cre-negative mice, indicating the effective inhibition of TGF-β signaling. Furthermore, overt OA-liked phenotypes were observed in TGFβRII^Col2ER^ mice, which was consistent with the previous reports (Jin et al., 2011). However, it was interesting that Osteoking seemed to lose its chondroprotective effect in TGFβRII^Col2ER^ mice. Accordingly, the expression of MMP-13 was still at a high level after Osteoking treatment. In general, according to the detection of TGFβRII deletion efficiency, there were still about 10–15% of TGFβRII remaining in the chondrocytes of TGFβRII^Col2ER^ mice and Osteoking was supposed to down-regulate the expression of MMP-13. These unexpected results suggest that Osteoking prevented cartilage from degradation partly through the TGF-β signaling pathway.

In conclusion, our study demonstrated that Osteoking could decelerate OA progression by preventing cartilage from degradation, reducing subchondral bone sclerosis, and improving gait disturbance. Moreover, we validated that the TGF-β signaling pathway played an important role in the positive effect of Osteoking on OA. However, more studies are needed to explore the more precise target genes of Osteoking. The mechanism of Osteoking in reducing subchondral bone sclerosis and ameliorating gait abnormality deserves further exploration.

## Data Availability

The raw data supporting the conclusions of this article will be made available by the authors, without undue reservation, to any qualified researcher.
